# **QTL** × **environment interactions underlie ionome divergence in switchgrass**

**DOI:** 10.1093/g3journal/jkab144

**Published:** 2021-04-29

**Authors:** Li Zhang, Alice MacQueen, Jason Bonnette, Felix B Fritschi, David B Lowry, Thomas E Juenger

**Affiliations:** 1 Department of Integrative Biology, University of Texas at Austin, Austin, TX 78712, USA; 2 Division of Plant Sciences, University of Missouri, Columbia, MO 65211, USA; 3 Department of Plant Biology and DOE Great Lakes Bioenergy Research Center, Michigan State University, East Lansing, MI 48824, USA

**Keywords:** allelic effects, antagonistic pleiotropy, bioenergy, conditional neutrality, differential sensitivity, GxE, ionome, QTLxE, reaction norm, switchgrass

## Abstract

Ionomics measures elemental concentrations in biological organisms and provides a snapshot of physiology under different conditions. In this study, we evaluate genetic variation of the ionome in outbred, perennial switchgrass in three environments across the species’ native range, and explore patterns of genotype-by-environment interactions. We grew 725 clonally replicated genotypes of a large full sib family from a four-way linkage mapping population, created from deeply diverged upland and lowland switchgrass ecotypes, at three common gardens. Concentrations of 18 mineral elements were determined in whole post-anthesis tillers using ion coupled plasma mass spectrometry (ICP-MS). These measurements were used to identify quantitative trait loci (QTL) with and without QTL-by-environment interactions (QTLxE) using a multi-environment QTL mapping approach. We found that element concentrations varied significantly both within and between switchgrass ecotypes, and GxE was present at both the trait and QTL level. Concentrations of 14 of the 18 elements were under some genetic control, and 77 QTL were detected for these elements. Seventy-four percent of QTL colocalized multiple elements, half of QTL exhibited significant QTLxE, and roughly equal numbers of QTL had significant differences in magnitude and sign of their effects across environments. The switchgrass ionome is under moderate genetic control and by loci with highly variable effects across environments.

## Introduction

Plants take up most of the elements of the ionome from soil, which is highly heterogeneousacross multiple spatial scales (Huang and Salt 2016). Studies in many plant species have examined the genetic architecture of the ionome and discovered strong genetic effects underlying elemental composition, and many quantitative trait loci (QTL) in genetic mapping experiments (Buescher *et al.* 2010; Lowry *et al.* 2012; Zhang *et al.* 2014; Shakoor *et al.* 2016). Studies in Arabidopsis *thaliana*, where transgenic manipulation is possible, have identified several causal genes controlling elemental variations (Rus *et al.* 2006; Morrissey *et al.* 2009; Chao *et al.* 2014). Recent work in *A. thaliana* has also shown signals of local adaptation to soil salinity, which could be driven by genetic loci that affect the ionome (Busoms *et al.* 2015). Regardless of plant species, studying genetic variation in the ionome can provide insights into how plants adapt to the highly variable soils that comprise the natural landscape, and can lead to the discovery of genes involved in elemental accumulation, including transporters, transcription factors, and metal-binding proteins (Rus *et al.* 2006; Baxter *et al.* 2008, 2010; Baxter and Dilkes 2012). However, previous work has provided limited insights into how the ionome varies in natural environments. The ionome of an individual depends not only on its genetic makeup, but also on the environment it experiences. Genetic variation in the makeup of the ionome between environments is a type of genotype-by-environment interaction (GxE).

The pattern of phenotypic expression of a single genotype across a range of environments is known as a *reaction norm*. Reaction norms make two important points about GxE explicit: first, that the phenotype expressed by a given genotype depends on the environmental context, and second, that the phenotypic effect in a given environment depends on the genotype in question (Gomulkiewicz and Kirkpatrick 1992). The reaction norm of a particular genotype and its underlying genetic architecture is heritable properties of the genome and can evolve. Alleles of a gene that affect a reaction norm can do so, and thus exhibit GxE, in multiple ways (Des Marais *et al.* 2013). For continuous phenotypes like elemental abundances, which have a given mean and standard deviation in two environments for a reference allele, the alternate allele of that gene can affect the magnitude or the sign of the phenotypic effect in one environment relative to the second. *Differential sensitivity* occurs when the magnitude of the phenotypic effect of an allele depends on the environment. *Conditional neutrality* is the most extreme case of differential sensitivity, which occurs when an allele affects the magnitude of the phenotype in one environment and not in another (Des Marais *et al.* 2013; El-Soda *et al.* 2014). *Antagonistic pleiotropy* occurs when the sign of the phenotypic effect of an allele depends on the environment (Kawecki and Ebert 2004; Des Marais *et al.* 2013; El-Soda *et al.* 2014). Studies of several biological systems in their natural environments have found that local adaptation is more often caused by conditional neutrality than antagonistic pleiotropy at the level of the QTL (Des Marais *et al.* 2013; Wadgymar *et al.* 2017).

To date, there has been limited progress in identifying the molecular mechanisms causing GxE in the plant ionome. GxE could not be examined in the many previous studies that identified ionomic QTL in a single environment (Loudet *et al.* 2007; Norton *et al.* 2010; Baxter *et al.* 2014; Zhang *et al.* 2014; Gu *et al.* 2015). These studies have largely focused on characterizing the elemental accumulation of various plant tissues or species, and though they have led to valuable knowledge on the genetic control of element accumulation in plants, they offer limited insights into how the ionome interacts with environment. More recently, studies have begun to identify GxE and QTL-by-environment interactions (QTLxE) for the plant ionome (Phuke *et al.* 2017; Veley *et al.* 2017; Ziegler *et al.* 2017; Fikas *et al.* 2019). These studies have been limited to biparental crosses or diversity panels with limited numbers of genotypes, particularly in short-lived, inbred crop species such as rice (*Oryza sativa*) and maize (*Zea mays*). Studies of GxE in the ionome in outbred, perennial systems may reflect different patterns of GxE, as these plants must cope with heterogeneous environments, including nonoptimal abundances of essential and nonessential elements, over their longer lifespans.

Switchgrass (*Panicum virgatum*) is an outbred, perennial species with wide environmental adaptation across the eastern half of North America and high biomass productivity across a large geographic range (Casler *et al.* 2007). Switchgrass was selected as a model bioenergy species by the U.S. Department of Energy in 1991 (Wright and Turhollow 2010), not only because of its high productivity across environments, but also its ecosystem services associated with carbon sequestration, soil erosion, and wildlife biodiversity (McBride *et al.* 2011). Switchgrass has substantial morphological diversity over its native range, including highly divergent southern lowland and northern upland ecotypes. The southern lowland ecotype of switchgrass is typically adapted to wet and riparian areas of the southern United States and tends to be more biomass-productive and nutrient-use-efficient than the northern upland ecotype (Porter 1966; Aspinwall *et al.* 2013; Uppalapati *et al.* 2013; Lowry *et al.* 2014). In contrast, the northern upland ecotype is often adapted to dry areas of mid and northern latitudes, and tends to be more freezing-tolerant (Hultquist *et al.* 1997; Casler 2012; Peixoto and Sage 2016). Ionomics research in switchgrass has identified significant differences in elemental uptake between lowland and upland ecotypes for many elements (Yang *et al.* 2009), including lower nutrient concentrations in lowland ecotypes; however, the genetic basis of this divergence has yet to be mapped. Nutrient elements are always removed along with harvested biomass; reduced nutrient removal necessitates lower fertilizer inputs to maintain plant productivity and thus promotes sustainable biofuel agriculture. High levels of some elements, particularly alkali metals, can negatively affect the downstream conversion to bioenergy and increase the cost of bioenergy production (Gouzaye *et al.* 2014; de Koff and Allison 2015; Serapiglia *et al.* 2016). However, marginal soils are likely to vary more in their elemental compositions than traditional arable land, making understanding GxE in the switchgrass ionome all the more essential to identify genes that can promote nutrient-efficient growth in these environments. Understanding the genetics of ionomic concentration divergence between switchgrass ecotypes across their native range will help breeders develop switchgrass as a sustainable biofuel species.

In this study, we expand the scope of GxE research in ionomics by evaluating the genetic architecture and reaction norms of the ionome in switchgrass. We use an outbred mapping population derived from a four-parent cross of lowland and upland ecotypes (Milano *et al.* 2016). We clonally propagated and planted the four parents, the two hybrid F_1_ genotypes, and approximately 750 F_2_ individuals at three common gardens, then quantified the accumulation of 18 elements. The 18 elements included macronutrients (Mg, P, K, and Ca), micronutrients (B, Mn, Fe, Co, Cu, Zn, Se, and Mo), analogs of macronutrients (Rb and Sr), and others that can be harmful to plant growth (Al, As, Cd) and that can be harmful or beneficial to plant growth (Na) (Marschner 2012). With these data, we evaluated the reaction norms of particular QTL for elements in the ionome. Our results allow us to address the following questions: (1) What is the genomic basis for variation in elemental abundances in the switchgrass ionome? (2) What fraction of QTL for distinct elements colocalize, suggesting possible common genetic architectures underlying their abundances? (3) How frequently do ionomic QTL show GxE? and (4) Which QTL colocalize with candidate genes, suggesting avenues for future molecular characterization of the switchgrass ionome?

## Materials and methods

### Experimental design and phenotyping

The details of the creation of the mapping population can be found in Milano *et al.* (2016). In brief, the genetic mapping population was produced from two initial crosses of two pairs of highly divergent southern lowland and northern upland ecotypes: lowland AP13 (A) × upland DAC6 (B), and lowland WBC3 (C) × upland VS16 (D). The F_1_ hybrids (A × B, C × D) were then intercrossed reciprocally to create a large full sib family that we utilize as a four-way linkage mapping population (F_2_).

The details of the experimental design are described in Lowry *et al.* (2019). Briefly, the grandparents, F_1_ hybrids, and the F_2_ progeny were propagated clonally in 3.8-L pots at the Brackenridge Field Laboratory, Austin, TX in 2013–2015, and then transported to and planted at the three field sites (Austin, Texas, hereafter TX; Columbia, Missouri, hereafter MO; and Hickory Corners, Michigan, hereafter MI) in May–July of 2015. Woven ground cover (Sunbelt 3.2 OZ, Dewitt Company) was used to suppress weeds, and holes were cut in a honeycomb fashion for planting of the experimental plants. Edge effects were prevented with a row of border plants. Plants were hand-watered as needed through the summer of 2015 to facilitate establishment, with no further supplemental irrigation after this point. Multiple replicates of the grandparent clones were grown at each site. However, our experimental design was unreplicated at the local field site level in terms of progeny; that is, we grew one single-spaced plant representing each progeny from the cross at each of the three common gardens, and these progeny were randomly arrayed across each common garden. The three common garden locations have distinct soil and climatic conditions. TX site (30.384°N, −97.73°W) has clay soil, MO (38.897°N, −92.22°W) common garden is located on a silt loam soil, and MI (42.420°N, −85.37°W) site has a loam soil. The concentrations of mineral P, K, Ca, Mg, Fe, Zn, Mg, Cu, Bo, and Na at each of the three sites were measured on a soil sample consisting of equally mixed proportions of soil samples (0–15 cm depth) from three locations spanning the entire garden on the diagonal. Soil samples were analyzed by the Soil, Water, and Forage Testing Laboratory at Texas A&M University (http://soiltesting.tamu.edu), and measurements of these minerals are presented in Table 2. The average temperatures in 2016 for TX, MO, and MI sites were 21.9, 13.6, 10.4°C, respectively. The annual precipitation in 2016 for TX, MO, and MI sites were 829, 928, and 975 mm, respectively.

**Table 2:** Element concentration (µg g^−1^) means ± standard errors of the outbred F_2_ mapping population, and comparisons by Welch one-way test at the three common gardens

	**Element** ^a^	TX garden	MO garden	MI garden	***P*-value** ^b^
Macronutrient	K	60,162 ± 882	60,032 ± 1010	55,912 ± 958	0.002*
	Soil K	246	144	63	CL: 125^c^
	Ca	3768 ± 35	1420 ± 12	1408 ± 15	<0.001*
	Soil Ca	23,596	3631	1476	CL: 180^c^
	Mg	1530 ± 14	1144 ± 8	1309 ± 11	<0.001*
	Soil Mg	262	448	150	CL: 50^c^
	P	421 ± 4	485 ± 7	294 ± 3	<0.001*
	Soil P	4	16	28	CL: 50^c^
Micronutrient	Mn	27.46 ± 0.31	80.63 ± 0.97	48.27 ± 0.58	<0.001*
	Soil Mn	4.62	19.90	40.32	CL: 1.00^c^
	Fe	43.48 ± 0.4	32.88 ± 0.41	27.69 ± 0.25	<0.001*
	Soil Fe	5.55	29.65	21.09	CL: 4.25^c^
	Zn	18.819 ± 0.349	10.995 ± 0.147	6.509 ± 0.096	<0.001*
	Soil Zn	0.93	0.48	0.52	CL: 0.27^c^
	Cu	4.926 ± 0.058	8.325 ± 0.117	3.801 ± 0.036	<0.001*
	Soil Cu	0.62	0.61	0.36	CL: 0.16^c^
	B	5.565 ± 0.059	2.645 ± 0.046	3.233 ± 0.06	<0.001*
	Soil B	1.03	0.31	0.22	CL: 0.60^c^
	Mo	0.053 ± 0.001	0.059 ± 0.001	0.032 ± 0	<0.001*
	Co	0.065 ± 0.001	0.14 ± 0.004	0.028 ± 0	<0.001*
	Se	0.047 ± 0.001	0.039 ± 0.001	0.009 ± 0.001	<0.001*
Analog	Sr	8.459 ± 0.073	8.534 ± 0.078	3.846 ± 0.04	<0.001*
	Rb	1.788 ± 0.027	2.436 ± 0.026	1.087 ± 0.019	<0.001*
Other	Na	70.46 ± 1.47	25.56 ± 0.53	9.72 ± 0.17	<0.001*
	Soil Na	14	24	8	
	Al	58.96 ± 0.73	76.17 ± 0.71	41.06 ± 0.5	<0.001*
	As	0.01 ± 0	0.013 ± 0	0.01 ± 0	<0.001*
	Cd	0.003 ± 0	0.024 ± 0.001	0.03 ± 0.001	<0.001*

a When the element indicated is prefaced by the word “Soil” the row contains average soil elemental concentration at this garden.

b Asterisks in this column indicate p-values that are significant after a Bonferroni correction for 18 independent Welch one-way tests.

c CL: Critical level. The point at which the Soil, Water, and Forage Testing Laboratory of Texas A&M University recommends no additional nutrient input.

Samples of developmentally staged phytomers (post-anthesis tillers) from the canopy of single-spaced plants (i.e., approximately 700 plants) were collected at each of the three sites at the end of the 2016 growing season, after approximately two years of growth in natural soils in each of the common garden. These tillers were dried and ground, then the ground tissue was sampled for ionomic analyses. Specifically, tiller samples were first ground with a knife mill (Wiley Model 4, Thomas Scientific) to pass through a screen size of 2 mm and subsequently ground with an inducted air abrasion mill (Cyclone Mill, UDY corporation) to pass through a 1 mm screen. The milled samples were homogenized and aliquots were sent to the Donald Danforth Plant Science Center to determine tissue concentrations of 18 elements (P, K, Ca, Mg, Rb, Sr, Mn, Zn, Cu, Co, Fe, Mo, B, Se, Al, Na, Cd, and As). Details of the process can be found in Ziegler *et al.* (2013). Briefly, tissue samples were weighed and digested in nitric acid at room temperature overnight, and then heated at 100°C for 3 hours. Elemental concentrations were measured by ICP-MS (Perkin Elmer NexION 350 D). Measurements were corrected for potential variation in sample preparation and instrument drift using both internal standards and matrix matched controls as described in Ziegler *et al.* (2013). Outliers and negative values yielded due to machine error were further excluded from analysis. Comparisons of elemental concentrations among the four grandparents at each common garden and comparisons of elemental concentrations of the F2 progeny among the three environments were performed using Welch one-way tests with a significance level of α = 0.05.

### Genotyping and map construction

Details on the genetic map construction can be accessed on https://datadryad.org/stash/dataset/doi:10.5061/dryad.ghx3ffbjv (Lovell *et al.* 2020) and in Bragg *et al.* (2020). In brief, Illumina fragment paired end libraries from each of the four grandparents were aligned to the *P. virgatum* reference genome v5 via bwa *mem* (Li and Durbin 2009) and used for single-nucleotide polymorphism (SNP) calling. Then a kmer-based approach was used to capture multiple variant and distinguish each grandparent when genotyping the progeny. The resulting genotype matrix was polished via sliding windows across the physical V5 switchgrass genome position and markers were re-ordered within linkage groups (Lowry *et al.* 2019; Lovell *et al.* 2020). Genotypes for progeny were based on grandparental haplotypes and thus are fully informative. For computational efficiency in GxE analysis, the genetic map was reduced to 738 markers, with an average distance of 2cM between markers.

### Heritability estimates and genetic correlation

We estimated quantitative genetic variation for the measured ionomic features within our full sib family using marker-based realized relationship matrices and linear mixed models implemented in the Sommer package (Covarrubias-Pazaran 2016) in R Core Team (2020). Due to potentially high correlation between the additive and dominance relationship matrices in a full sib family, it was not feasible to cleanly partition additive from nonadditive components of variance (Hill 2013). As such, our analyses based on the additive kinship matrix alone could be biased upwards by any dominance variance which occurs. We thus report our estimates from the additive kinship matrix as genetic variance (*V_g_*), and our heritabilities as broad-sense heritability (*H^2^*), which was calculated as *V_g_/V_p_*, where *V_p_* is the total phenotypic variance. For genetic correlation estimates, combinations of phenotypic data from the three sites were used as response variables in the multivariate model for each ionomic trait.

We further tested for GxE on the trait level using the same mixed model approach (Covarrubias-Pazaran 2016, https://cran.r-project.org/web/packages/sommer/vignettes/v4.sommer.gxe.pdf, last accessed in Aug, 2020). In other words, we tested whether *V_g_* differed by site for each element. Specifically, we used a likelihood-ratio test to compete two models. The first model (*i.e.*, main effect model) assumed that there is no GxE and that the inclusion of two parameters, the genetic variance plus the fixed effect for environment, was sufficient for modeling the data. The alternative model (*i.e.*, unstructured model) also accounts for GxE, and additionally freely estimates a unique genetic variance and covariance (a 3 × 3 unstructured variance-covariance matrix) within and across environments. Significance of the likelihood-ratio test for GxE was assessed at the level of *α *= 0.05.

### Multi-environment QTL mapping

Details of the mapping procedures and implementation for the four-way population using Genstat are described in Malosetti *et al.* (2013), Lowry *et al.* (2019), and Bragg *et al.* (2020). Specifically, we used the “single trait under multiple environments” multi-environment mixed model for each ionomic element for a cross-pollinated (CP) families as implemented in Genstat v.19 (VSN International 2020). Our experimental population contained four possible QTL alleles: those designated *A* and *B* corresponded to marker alleles of the first pair of grandparents (AP13 and DAC), and those designated *C* and *D* corresponded to marker alleles of the second pair of grandparents (WBC and VS16). The initial step for QTL mapping using Genstat was to identify the best variance-covariance matrix model for the phenotypic data (Malosetti, *et al.* 2013). Subsequently, simple interval mapping (SIM) was performed for a preliminary scan of the genome using the 738 markers (genetic predictors). The identified QTL were then used as cofactors in a follow-up composite interval mapping (CIM) scan. QTL scanning was performed with a window size of 5 cM and 50 cM was used as the minimum cofactor distance in the CIM scans. CIM was performed three times consecutively to ensure the stability of identified QTL in our study.

QTL identified through CIM were simultaneously incorporated into a mixed effect model with the variance-covariance matrix selected for the trait of the form:

(1) $$\mathrm{trait}=µ+E+\sum \mathrm{QTL}+\sum \left(\mathrm{QTLxE}\right)+e$$

where μ represents the population mean; *E* represents the environment effect; $\sum \mathrm{QTL}=\sum \left(a^{a1}+a^{a2}+a^{d}\right)$, represents the total effect from the additive effect from the first grandparent [*i.e.*, the difference between *A* (AP13) and *B* (DAC) alleles], $a^{a1}$, the second grandparent [*i.e.*, the difference between *C* (WBC) and *D* (VS16) alleles], $a^{a2}$, and the dominance effect [*i.e.*, the intralocus interaction], $a^{d}$; $\sum \left(\mathrm{QTLxE}\right)$represents the QTL × environment interactions; and *e* represents the error term. QTL significance was assessed using the Wald test statistic, and the final model was selected using a backward selection procedure based on the Akaike’s Information Criterion (AIC, Akaike 1974). Genome-wide QTL and QTL × E significance were assessed at α  =  0.05 with a Bonferroni correction (Li and Ji 2005). QTL were localized based on a 1.5 LOD statistic drop from the highest LOD score, and used the flanking markers associated with a 1.5 LOD statistic drop from the peak as the confidence interval for the QTL peak.

### Candidate gene search and GO enrichment analyses

We consider the genes located in the 1.5-LOD confidence intervals around the detected significant QTL as candidate genes. We then determined if homologs from rice (v7), *A. thaliana* (TAIR 10), and a curated list of genes that affect the plant ionome (Whitt *et al.* 2020) were overrepresented in our QTL regions. The annotation file for switchgrass was accessed on JGI (Joint Genome Institute) Phytozome 13 website: https://njp-spin.jgi.doe.gov/ (last accessed in Aug, 2020). The Gene Ontology (GO) enrichment analysis was conducted using Fisher’s exact test for each GO term via R package “topGO” (Alexa and Rahnenfuhrer 2020). GOs with adjusted *P *<* *0.05 were considered significant.

### Data availability

The supplemental materials are available at figshare: https://doi.org/10.25387/g3.14479185.

## Results

### The genetic basis of elemental concentration variation and covariation at three common gardens

To understand the genetic component of ionomic variation in switchgrass, we determined concentrations of 18 elements for both the F_0_ “grandparent” genotypes and for the outbred F_2_ genotypes at three common gardens. Average concentration varied over six orders of magnitude among elements across environments: Co, Se, Mo, and Cd had the lowest concentrations (∼1 × 10^−2 ^µg g^−1 ^dry weight) and K had the highest concentration (∼1 × 10^4^ µg g^−1 ^dry weight). After correction for multiple testing, concentrations of 11 of the 18 elements differed significantly between the four grandparents (AP13, DAC6, WBC, and VS16) at one or more gardens (Welch one-way test, Table 1). Concentrations of three elements (Ca, P, and Na) differed significantly between the four grandparents at every garden after correction for multiple testing, and Sr and Mg concentrations also differed at every garden before this correction (Welch one-way test, Table 1). Interestingly, there were just as many significant differences in element*garden concentrations (16) between the two lowland genotypes, AP13 and WBC, as there were between the upland and lowland parents. In contrast, there were only two significant differences in element*garden concentrations between the two upland parents (data not shown).

**Table 1:** Element concentration (µg g^−1^) means, standard errors, and comparisons by Welch one-way test of the four F_0_ “grandparent” individuals at the TX, MO, and MI gardens

	Element	Site	AP13	DAC	VS16	WBC	***P*-value** ^a^
macronutrient		MI	72,581 ± 3741	46,184 ± 1711	31,615 ± 3024	66,643 ± 12,666	<0.0001*
	K	MO	54,865 ± 5417	44,609 ± 11,478	24,143 ± 8032	83,190 ± 10,820	0.0419
		TX	54,414 ± 5221	59,728 ± 13,856	39,167 ± 5242	67,527 ± 7067	0.0525
		MI	1614 ± 48	2046 ± 102	1163 ± 48	1454 ± 123	<0.0001*
	Ca	MO	1445 ± 47	1395 ± 80	1101 ± 24	1736 ± 155	0.0002*
		TX	2947 ± 149	5293 ± 362	3953 ± 156	2168 ± 82	<0.0001*
		MI	1367 ± 50	1011 ± 73	1059 ± 50	1686 ± 112	<0.0001*
	Mg	MO	857 ± 25	767 ± 47	784 ± 50	1497 ± 117	0.0175
		TX	949 ± 55	1333 ± 101	1154 ± 42	1027 ± 52	0.0182
		MI	296 ± 10	391 ± 21	386 ± 18	441 ± 24	<0.0001*
	P	MO	615 ± 41	378 ± 43	346 ± 5	851 ± 39	<0.0001*
		TX	316 ± 12	758 ± 53	650 ± 41	300 ± 16	<0.0001*
micronutrient		MI	47.3 ± 2.14	52.22 ± 3.88	53.39 ± 3.76	33.605 ± 2.882	0.0009
	Mn	MO	67.04 ± 3.74	70.9 ± 7.88	101.45 ± 24.06	76.523 ± 7.952	0.5783
		TX	25.56 ± 1.49	39.85 ± 3.61	38.86 ± 3.17	14.212 ± 1.221	<0.0001*
		MI	32.33 ± 1.21	41.7 ± 3.58	34.27 ± 1.84	30.199 ± 1.448	0.0458
	Fe	MO	39.64 ± 2.4	83.06 ± 52.69	32.4 ± 1.78	45.761 ± 6.237	0.1069
		TX	51.5 ± 2.75	78.42 ± 12.89	50.78 ± 7	44.089 ± 4.489	0.1662
		MI	7.51 ± 0.934	7.54 ± 0.406	11.39 ± 2.796	8.136 ± 1.636	0.6080
	Zn	MO	22.43 ± 3.802	11.36 ± 0.912	11.58 ± 0.898	28.504 ± 10.996	0.0754
		TX	49.34 ± 13.966	110.91 ± 86.947	15.75 ± 2.458	18.849 ± 1.185	0.1489
		MI	3.223 ± 0.144	5.333 ± 0.261	4.919 ± 0.125	3.332 ± 0.164	<0.0001*
	Cu	MO	8.715 ± 0.538	12.848 ± 4.019	8.03 ± 0.291	9.919 ± 0.836	0.1985
		TX	4.205 ± 0.229	6.152 ± 0.727	4.141 ± 0.403	5.094 ± 0.378	0.0729
		MI	3.417 ± 0.247	4.12 ± 1.188	3.294 ± 0.431	3.32 ± 0.502	0.9330
	B	MO	3.402 ± 0.704	3.196 ± 0.673	3.319 ± 2.247	2.476 ± 0.273	0.6658
		TX	4.925 ± 0.421	7.211 ± 0.432	6.852 ± 0.537	4.402 ± 0.319	0.0005*
		MI	0.046 ± 0.002	0.039 ± 0.003	0.051 ± 0.003	0.041 ± 0.003	0.0603
	Mo	MO	0.087 ± 0.004	0.056 ± 0.005	0.053 ± 0.015	0.122 ± 0.009	0.0143
		TX	0.092 ± 0.011	0.044 ± 0.005	0.053 ± 0.007	0.117 ± 0.018	0.0004*
		MI	0.029 ± 0.002	0.066 ± 0.016	0.046 ± 0.007	0.026 ± 0.004	0.0356
	Co	MO	0.219 ± 0.057	0.321 ± 0.186	0.145 ± 0.025	0.168 ± 0.036	0.6059
		TX	0.082 ± 0.008	0.149 ± 0.047	0.189 ± 0.122	0.11 ± 0.033	0.4476
		MI	0.01 ± 0.004	0.012 ± 0.004	0.007 ± 0.002	0.041 ± 0.003	0.1384
	Se	MO	0.042 ± 0.003	0.05 ± 0.017	NA	0.122 ± 0.009	0.1384
		TX	0.044 ± 0.004	0.048 ± 0.01	0.038 ± 0.006	0.117 ± 0.018	0.1384
		MI	3.831 ± 0.14	5.834 ± 0.977	3.258 ± 0.201	3.709 ± 0.333	0.0418
analog	Sr	MO	9.093 ± 0.575	8.81 ± 0.768	6.27 ± 0.221	9.684 ± 0.899	0.0011
		TX	6.362 ± 0.263	8.866 ± 0.287	9.502 ± 0.482	5.601 ± 0.231	<0.0001*
		MI	1.509 ± 0.084	0.966 ± 0.112	0.728 ± 0.07	3.026 ± 0.284	<0.0001*
	Rb	MO	2.923 ± 0.162	1.245 ± 0.129	0.94 ± 0.036	3.719 ± 0.222	<0.0001*
		TX	1.565 ± 0.123	1.5 ± 0.305	1.451 ± 0.21	2.079 ± 0.203	0.1951
		MI	50.5 ± 3.48	8.67 ± 1.64	12.71 ± 4.98	47.892 ± 6.147	<0.0001*
Other	Na	MO	160.83 ± 7.53	11.87 ± 1.43	10.08 ± 1.31	59.685 ± 7.239	<0.0001*
		TX	122.87 ± 12.37	35.46 ± 5.04	65.56 ± 14.28	124.885 ± 15.271	<0.0001*
		MI	48.79 ± 2.46	69.19 ± 14.38	59.73 ± 5.04	49.204 ± 3.266	0.1845
	Al	MO	102.17 ± 10.24	95.78 ± 30.36	77.56 ± 10.51	84.231 ± 5.996	0.5187
		TX	68.36 ± 5.2	100.48 ± 16.74	77.55 ± 7.45	56.923 ± 4.699	0.0656
		MI	0.01 ± 0.001	0.019 ± 0.004	0.012 ± 0.001	0.011 ± 0.001	0.1384
	As	MO	0.016 ± 0.003	0.022 ± 0.017	NA	0.022 ± 0.003	0.1384
		TX	0.011 ± 0.001	0.017 ± 0.005	0.012 ± 0.001	0.01 ± 0.001	0.1384
		MI	0.016 ± 0.001	0.022 ± 0.002	0.012 ± 0.001	0.013 ± 0.002	0.0027
	Cd	MO	0.03 ± 0.011	0.028 ± 0.01	0.015 ± 0.006	0.017 ± 0.002	0.6142
		TX	0.002 ± 0	0.003 ± 0	0.002 ± 0	0.002 ± 0	0.0216

a Asterisks in this column indicate *P*-values that are significant after a Bonferroni correction for 54 independent Welch one-way tests.

**Figure 1:**
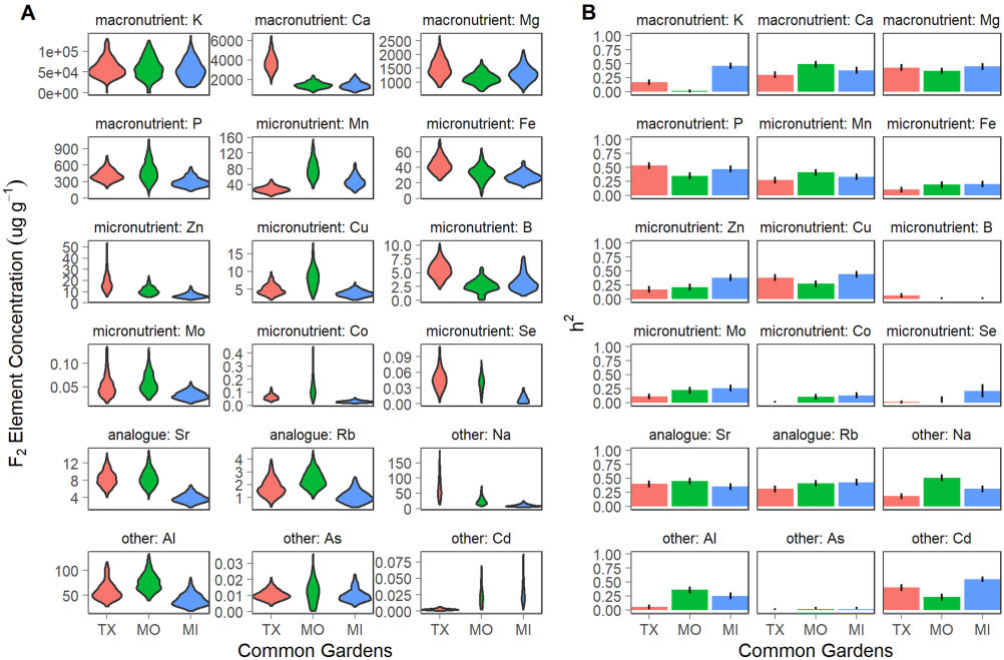
The genetic component of phenotypic variation in element concentrations (µg g^−1^) across three common gardens (TX: orange; MO: green; MI: blue) (A) Phenotypic variation in element concentrations for the mapping population (F_2_). (B) Heritability of each element concentration.

In the F_2_ genotypes, variation in the concentration of each element followed a continuous, unimodal distribution within each garden (Figure 1A). Within gardens, the majority of the element concentrations were not strongly phenotypically correlated (*r* < 0.5); fewer than 3% of element pairs had positive correlations greater than 0.5 (Supplementary Table S1). Among these, Ca concentration was positively correlated with Sr concentration at each site (0.8–0.9), and Al concentration was positively correlated with Fe concentration at MI (0.8) and TX (0.5).

All element concentrations had low to moderate broad sense heritabilities (0 < *H*^2^ < 0.6, Figure 1B). The majority of the elements (K, Ca, Mg, P, Mn, Fe, Zn, Cu, Mo, Se, Sr, Rb, Na, Al, and Cd) had moderate heritabilities (0.2 < *H^2^* < 0.6) for at least one garden, while B, Co, and As had low heritabilities (*H*^2^ < 0.2) everywhere. There were moderate heritabilities for 8 elements in the TX garden (none unique to TX), 12 elements at the MO garden (Na and Al concentration were moderately heritable only at MO), and 15 elements at the MI garden (K, Zn, Se, and Cd concentration were moderately heritable only at MI). The low heritabilities of some elements at certain sites (B, K, Co, As, and Se) were due to both the large error variance (*V_e_*) and the near zero genetic variance (*V_g_*) for the concentrations of these elements (Supplementary Table S2). Likelihood-ratio tests between models with genetic effects only and models with genetic and GxE effects indicated that GxE existed for 16 of the 18 elements (all but B and Se) at the trait level (*P *<* *0.05). Thus, switchgrass exerted genetic control of elemental accumulation in an environmentally sensitive fashion for the majority of the elements of the ionome.

The distributions of all 18 element concentrations also differed significantly among gardens (all *P *<* *0.002, Welch one-way tests, Table 2). These distinct phenotypic distributions were undoubtedly affected by soil element concentrations and availability, which varied in ways that affected plant element concentrations in both intuitive (Ca and K) and nonintuitive (Mg, P, and Na) fashions (Table 2). They were also underlain by moderate to strong positive genetic correlations for the majority of the elements among gardens (Supplementary Table S3). Positive genetic correlations less than one indicate the presence of GxE at the trait level, and likely magnitude-changing instead of sign-changing patterns of GxE at the level of QTL across the common gardens for the elemental concentrations. Only one negative genetic correlation was observed, for B concentration in the TX and MO gardens (−0.46). Negative correlations indicate a possible trade-off in loci controlling B concentration. It should be noted, however, that B concentration heritabilities were low at both of these gardens, reducing our power to identify QTL. The genetic correlations for two elements (As and Se) could not be determined because the concentrations of these elements had close to zero genetic variance.

**Figure 2:**
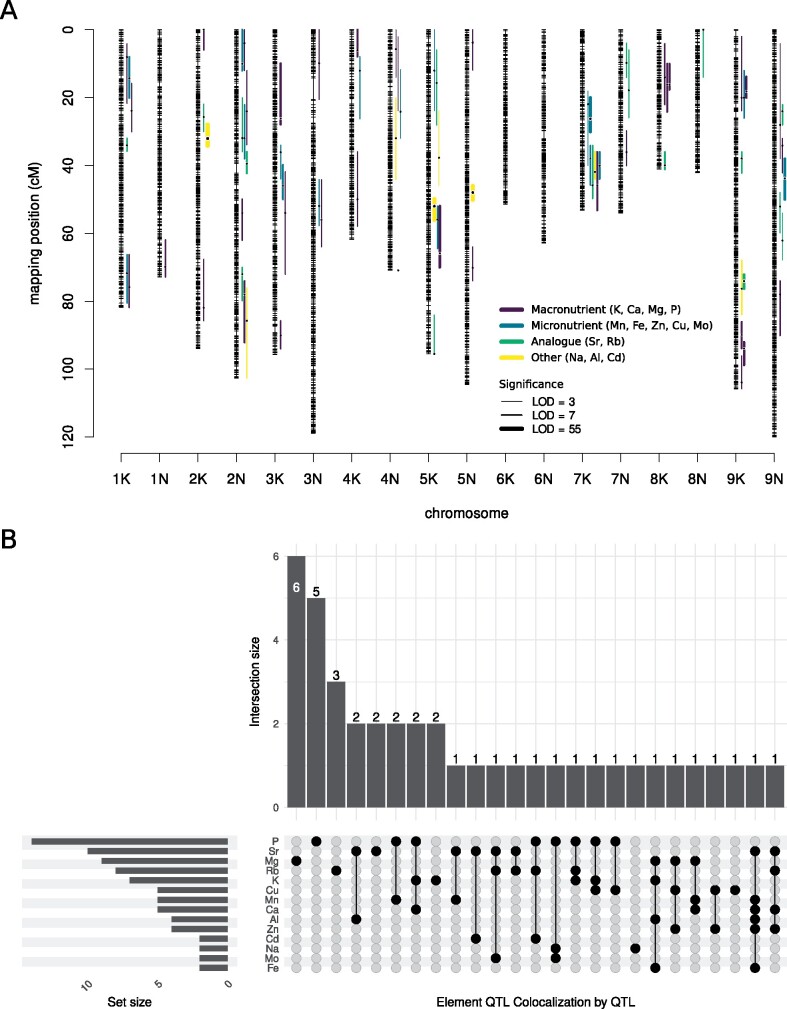
The overlapping genomic distributions of QTL for element concentrations (µg g^−1^). (A) QTL with 1.5-LOD supportive intervals for each ionomic trait using the multi-environment QTL model from Genstat. Purple indicates macronutrient QTL, blue micronutrient QTL, green analog QTL, and yellow other QTL. Further discrimination between QTL for each element within these categories is included in Supplementary Figure S1. (B) UpSet plot showing patterns in elemental concentration QTL colocalization between elements. Vertical barplot shows the number of QTL that colocalize for each combination of elements, represented by the filled circles connected by lines when more than one element colocalizes. Horizontal barplot shows the number of QTL for each element.

We next identified QTL and QTLxE interactions using independent multi-environment mixed models for each of the 18 elements. We detected 77 significant QTL with LOD thresholds above 3.5 for concentrations of 14 elements (Figure 2a, by category; Supplementary Figure S1, within category and Supplementary Table S4 for QTL position, LOD statistics, and so on). Thirty-eight (49%) of these QTL exhibited QTLxE (Supplementary Table S4). No significant QTL were detected for B, As, Co, and Se, almost certainly because of the low heritabilities of the tissue concentrations of these four elements (Figure 1B). The remaining elements had between two (Na, Fe, Mo, Cd) and 14 (P) significant QTL. We determined if the number of QTL we identified varied by element type by dividing the 18 elements into four types: macronutrients, micronutrients, nonessential analogs to nutrients, and other nonessential elements. The presence of more elemental QTL in a category than expected indicates ecotype-specific genetic divergence, while the presence of fewer than expected might indicate that purifying selection has removed genetic variation for these elements. If QTL had been equally distributed across the elements, we would have expected 17, 34, 8, and 17 QTL in these classes, respectively. However, there were more QTL than expected for both macronutrients (2.05x, binomial test *P *<* *0.001) and nonessential analogs (1.99x, binomial test *P *=* *0.002), and fewer QTL than expected for micronutrients (0.50x, binomial test *P *<* *0.001) and other nonessential elements (0.47x, binomial test *P *=* *0.013).

### QTL colocalization across elements of the ionome

Using our 77 QTL, we next identified QTL where distinct elements colocalized. Co-localization suggests either linked genes affecting element accumulation, or may indicate co-transport of elements using the same channel. The latter is more plausible for elements that are most commonly bioavailable in the soil as similar ions. We considered QTL colocalizing if there was any overlap in the genomic region with LODs within 1.5-LOD of the maximum LOD score. Twenty-one sets of QTL colocalized, and 20 QTL (26.0%) did not overlap another ionomic QTL, and hence were singletons (Figure 2B). Mg was the only element with a majority of singleton QTL, with both more noncolocalizing and fewer colocalizing QTL than expected (chi-square test, *P *=* *0.005). P had the most colocalizing QTL. Colocalizing P QTL always colocalized with elements which are most abundant in soil as cations with 1+ or 2+ charge. Ca QTL always colocalized, either with P (2 QTL) or with elements most abundant in soil as 2+ or 3+ cations (3 QTL). Al QTL is also always colocalized, with Sr in 3 of 4 QTL, and with Fe for both Fe QTL. The partial colocalization of QTL between Ca and Sr, and between Al and Fe, may underlie some of the high phenotypic correlation in these traits in the F_2_ genotypes (Supplementary Table S1). Three QTL sets colocalized four or more elements. One of these sets was located at 6.63–33.56 Mb on Chr02N with Ca, Zn, Rb, and Sr QTL, one at 0.97–41.75 Mb on Chr04N that included Mg, K, Fe, and Al QTL, and the third at 33.91–51.66 Mb on Chr07K that included Al, Ca, Mn, Fe, Zn, and Sr QTL (Figure 2A).

### Ionomic QTLxE frequencies and QTL reaction norms

**Figure 3:**
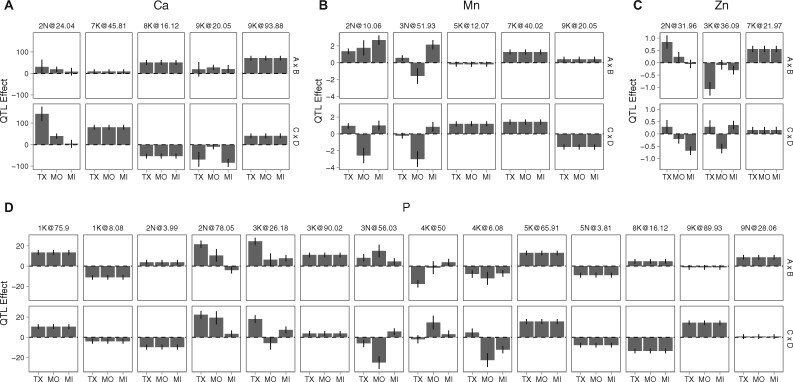
Representative differentially sensitive and antagonistically pleiotropic reaction norms for element concentrations (µg g^−1^) additive QTL effects across three common gardens (TX, MO, and MI). Two allelic contrasts are shown: panels A and B show QTL effects for the lowland AP13 × upland DAC cross, and panels C × D show QTL effects for the lowland WBC × upland VS16 cross. (A) Ca (macronutrient): 2 N@24.04 shows differential sensitivity in both allelic contrasts. (B) Mn (micronutrient): 2 N@10.06 shows differential sensitivity in one allelic contrast, (C) Zn (micronutrient): 3 K@36.09 shows antagonistic pleiotropy in one allelic contrast, and (D) P (macronutrient): has five QTL with QTLxE with distinct reaction norms at each QTL.

We next explored patterns of effect sizes, and types of QTLxE, in the 77 QTL, particularly the 38 QTL exhibiting QTLxE (Figure 3 and Supplementary Figure S2). The design of the crosses that generated the four-way population also allowed quantification of differences in allelic effects for two distinct lowland *vs* upland crosses, AP13 *vs* DAC (A × B) and WBC *vs* VS16 (C × D). In addition to looking at patterns of GxE within these crosses, we could also determine if we had captured variation in effects between these crosses, for both QTL with and without QTLxE effects. For the 39 QTL without QTLxE, most effects (75%) had the same effect direction in both lowland *vs* upland contrasts (Supplementary Figure S2). Thus, most QTL without QTLxE exhibited differences in QTL effects between the upland and lowland sets of parents, and few exhibited differences in QTL effects between the two upland or the two lowland parents. Of the 10 QTL without QTLxE but with within-ecotype variation, two QTL were singletons, and four colocalized with elements which had no significant QTLxE. The remaining four QTL colocalized with elements which did have QTLxE. These four QTL may well be caused by multiple linked loci; however, if these four colocalizing QTL are due to single loci that affect the concentration of multiple elements, then these QTL represent an interesting case of GxE caused by changes in pleiotropy at a single locus.

For the 38 QTL, and 76 allelic contrasts with QTLxE, 35 contrasts (46%) had differential sensitivity in their reaction norm across gardens, and 15 of these contrasts were statistically significant after a multiple testing correction (Bonferroni *t*-test, *P *<* *0.000198, Supplementary Figure S2). These differentially sensitive effects were observed in either one or both lowland *vs* upland allelic contrasts for the same QTL. For instance, the effect of QTL 2N@24.04 for the macronutrient Ca was differentially sensitive in both allelic contrasts (Figure 3A), while the effect of QTL 2N@10.06 for the micronutrient Mn was differentially sensitive only in the A × B contrast (Figure 3B). The other 41 allelic contrasts (54%) exhibited antagonistic pleiotropic effects (*i.e.*, a sign change) across gardens, and 13 of these contrasts were statistically significant after a multiple testing correction (*t*-test, *P *<* *0.000198, Supplementary Figure S2). The majority of the antagonistic effects were present in only one allelic contrast. For example, the effects of QTL 3K@36.09 for the micronutrient Zn were antagonistic for the C × D contrast, but not the A × B contrast (Figure 3C). Overall, QTL for the same element with QTLxE did not have similar patterns across environments. For example, the QTL 2N@78.05 and 3K@26.18 for the macronutrient P had the largest effects in TX, while the other two QTL 3N@56.03 and 4K@6.08 for P had the largest effect in MO (Figure 3D).

Our QTL mapping strategy allowed us to test for both additive parental effects and for intralocus interaction (dominance) between additive effects (Equation 1). Of the 77 detected QTL, 15 (19%) have significant dominance terms, and half of these showed dominance by environment interactions (Supplementary Table S5). The majority of the intralocus interaction effects were complex, with only a few (4) showing clear upland or lowland dominance patterns. In general, dominance effects were small relative to additive effects (9.80% on average).

### Ionomic QTL colocalization with candidate genes

To explore avenues for future molecular characterization of the switchgrass ionome, we determined the genetic content of the 77 QTL intervals for genes and gene ontology (GO) terms. We first examined QTL colocalization with candidate genes from ionomic mapping studies in other plant species, and found six important candidate genes (Supplementary Table S6) in the QTL intervals affecting element concentration in switchgrass. For example, *Pavir.9NG231800*, a homolog of *MOT1*, is located within the 1.5-LOD interval of the largest Mo concentration QTL (Chr09N@43.81). *MOT1*, which encodes a molybdate transporter, is responsible for the natural variation in Mo accumulation in *A. thaliana* and in rice (Baxter *et al.* 2008; Huang *et al.* 2019), and may play an important role in adaptation to acidic soils (Poormohammad Kiani *et al.* 2012). *Pavir.7kg416470*, a homolog of *HKT1*, was a candidate gene in the QTL interval on Chr07K which colocalized for six elements. *HKT1* encodes a Na transporter, and is responsible for the variation of Na content in *A. thaliana* (Rus *et al.* 2006; Baxter *et al.* 2010), rice (Ren *et al.* 2005), and wheat (Munns *et al.* 2012). Interestingly, this candidate gene was in the QTL interval for Al, Ca, Fe, Mn, Sr, and Zn, and did not contain a QTL for Na concentration in our mapping population. Candidate genes for heavy metal-associated ATPases, which are homologs of *HMA* in *A. thaliana* and rice, were found in Cu (Chr01K@14.42 and Chr07K@26.27), Cd (Chr02N@85.72), and Zn (Chr02N@71.96) QTL intervals. These genes are responsible for Cu, Cd, and Zn transport. A sixth candidate gene, *Pavir.9KG014451*, was associated with the homolog of *A. thaliana MYB36*. *MYB36* is a MYB domain transcription factor that regulates the expression of genes involved in the formation of the Casparian strip. The absence of the Casparian strip results in changes in leaf concentrations of Na, Mg, Zn, Ca, Mn, and Fe in *A. thaliana* (Kamiya *et al.* 2015). This candidate gene was in the QTL colocalizing Ca (Chr09K@20.05), Mg (Chr09K@18.15), and Mn (Chr09K@20.05) concentrations.

To elucidate the cellular pathways associated with ion concentrations in switchgrass, we also looked at GO term enrichment based on the gene content in the 77 QTL. We identified 405 unique enriched GO terms across the ionomic traits (*P *<* *0.05). Overall, these QTL regions were enriched for GO terms of DNA-binding transcription factor activity, heme binding, and oxidoreductase activity (Supplementary Table S7). Among the macronutrients and analogs of macronutrients, the QTL regions of Mg were significantly enriched for GO terms of carbohydrate binding, protein transport, cell wall biogenesis, and signal peptide processing, among the 34 ontologies. Mg is involved in protein synthesis (approximately 75% of leaf Mg), is associated with chlorophyll (15–20% of total Mg), and functions as a cofactor for a series of enzymes involved in photosynthetic carbon fixation and metabolism (Cakmak and Kirkby 2008; White and Broadley 2009). K QTL regions were significantly enriched for GO ontologies of oxidoreductase activity, calcium and iron ion binding, and in particular, antioxidant activity. K has a regulatory function in several biochemical processes related to protein synthesis, carbohydrate metabolism, and enzyme activation. K can enhance antioxidant defense in plants, which protects plants from oxidative stress in adverse environments (Hasanuzzaman *et al.* 2018).

Among the micronutrients, Mn concentration QTL intervals were significantly enriched for GO ontologies of photosynthesis, mitochondria, carbohydrate binding, the photosystem I reaction center, and electron transfer activity. Mn functions as a major contributor to various biological systems including photosynthesis, respiration, and nitrogen assimilation in plants among other functions (Andresen *et al.* 2018; Alejandro *et al.* 2020). Cu concentration QTL regions were significantly enriched for GO ontologies of cell wall macromolecular catabolic process, oxidoreductase activity, calcium ion binding, and regulation of transcription among the 36 ontologies. Cu is an essential cofactor for numerous proteins, an essential player in electron transport. Cu is also involved in the control of cellular redox state (a major Cu-binding protein is the Cu/Zn superoxide dismutase) and remodeling of the cell wall (Cohu and Pilon 2010; Andresen *et al.* 2018). Among nonessential elements, Cd QTL regions were significantly enriched for GO ontologies of metal ion binding, photosynthesis (light harvesting), and cell growth among others. Cd is one of the most toxic heavy metals for plants and can displace essential metals (such as Zn, Fe, and Ca) from a wealth of metalloproteins and disturb normal physiological processes. It can also cause severe developmental aberrance such as chloroplast structure change, reactive oxygen species (ROS) production, and cell death (Wan and Zhang 2012).

## Discussion

Ionomics is a powerful tool for determining the elemental status of plants, and can be combined with mapping populations to determine the genetic architecture responsible for variation in elemental composition. Our study not only examined the genetic basis of the switchgrass ionome, but also how individual ionomic loci responded to three environments (*i.e.*, expressed GxE) across the native range of this perennial species. We detected 77 significant QTL across the 18 elements, half of which had significant QTLxE effects. This indicated the importance of the environmental context in elemental concentration variation at the QTL level. We observed common QTL colocalization between elements, which supports a partially shared regulatory network for element uptake, transportation, or accumulation, as previously suggested (Baxter *et al.* 2014; Dhanapal *et al.* 2018). Understanding the genetic architecture of elemental accumulation in our outbred population of divergent switchgrass ecotypes is the first step in uncovering the potential for ionomic adaptation in switchgrass across variable environmental conditions.

Genotype by environment interactions are common across many different species, phenotypes, and environments. Previous work has found that GxE is often caused by differential sensitivity in response to the environment, and that antagonistic pleiotropy (or trade-offs) at the individual gene level are relatively rare or weak (Des Marais *et al.* 2013; Wadgymar *et al.* 2017; Lowry *et al.* 2019). Our study found not only differentially sensitive effects, but also substantial antagonistic pleiotropy (54%) across the ionomic QTL with QTLxE, indicating that alleles commonly had opposing effects on element concentrations in different environments. This result suggests that the plant ionome may play an important role in local adaptation, as both model and empirical work have suggested that there should be strong trade-offs involved in local adaptation at the level of QTL (Felsenstein, 1976; Bradshaw and Schemske 2003; Kawecki and Ebert 2004). Our cross design also allowed us to compare allelic effects for two distinct lowland *vs* upland crosses and determine if there was variation in effects between these crosses. Interestingly, some ionomic QTL showed differential sensitivity in one cross but antagonistic pleiotropy in the other. This suggests that the same set of loci may not be consistently responsible for divergence between lowland and upland switchgrass ecotypes, and implies that substantial ionomic variation also exists within upland and lowland ecotypes. In essence, these results suggest that different loci contribute to ionomic variation across the range of the species, and that ionomic divergence among ecotypes was not based on fixed differences between the ecotypes.

QTL for multiple elements typically colocalized in our study. This may not be surprising, as maintaining ion homeostasis requires a network of ion uptake, transportation, trafficking, and sequestration mechanisms, and not all genes in this regulatory network will be ion-specific (Clemens 2001). We found substantial colocalization of P QTL with cation QTL, always with elements most abundant in soil as cations with 1+ or 2+ charge. P is a component of key molecules of plants such as ATP, nucleic acids, and the form of P most readily accessed by plants, inorganic P, is likely co-transported with positively charged ions (Schachtman *et al.* 1998). Colocalization of P QTL with cation QTL in our study might thus reflect co-transport of P and cations at the gene level. Indeed, we found a few cation transporters annotated for *A. thaliana* in the P QTL intervals, including high-affinity K+ transporter, ZIP metal ion transporter family, and Ctr copper transporter family. P QTL colocalized with K and/or Ca QTL at three positions (8 K@10.7, 9 K@60.9, and 9 N@2.4). P, K, and Ca are all macronutrients, which plants need in large quantities. Although different populations may have adapted to soil types with different quantities of these elements, the need for these macronutrients in large quantities could have facilitated the evolution of similar or shared mechanisms or networks to take up these elements from soils, thus yielding colocalizing QTL. Alternatively, colocalization could be coincidental and/or simply due to multiple linked genes. In support of this view, P also had many QTL that were singletons (5 noncolocalizing QTL out of 14), as did the macronutrient Mg (6 noncolocalizing QTL out of 9). P and Mg deficiencies in soils are often widespread (Maathuis 2009); thus, a potential adaptive scenario is that switchgrass plants were under stronger selection to increase uptake or tolerate lower levels of accumulation of these two macronutrients, the segregation of which drove the increase in variation for concentrations of these elements and led to ion-specific QTL. Indeed, our study identified significantly more QTL for macronutrients than expected (2.05x enrichment, binomial test *P *<* *0.001). Identification of these QTL and their reaction norms is the first step in testing hypotheses of local adaptation in natural environments.

We detected fewer QTL than expected for micronutrients (0.5x, binomial test *P *<* *0.001), and most micronutrient QTL colocalized with QTL of other elements. Taken together, these results suggest that there may have been only weak selection on accumulation of micronutrients in switchgrass populations. It is possible that switchgrass obtains sufficient quantities of these micronutrients from any soil. This may be consistent with a recent study of the influence of Mn availability on switchgrass biomass production, showing that even low shoot tissue Mn allows switchgrass to maintain biomass production (Guo and Fritschi 2021). We also found little variation in concentration of potentially harmful elements (Al, As, and Cd), and fewer QTL than expected for these elements (0.47x, binomial test *P *=* *0.013). It may be that harmful elements impose such strong selection that beneficial alleles have been fixed, and deleterious alleles purged, at least in the populations from which the four grandparents were sampled. Alternatively, harmful elements may not be present in sufficient quantities in the commonly encountered soils and in the three common garden soils for the four grandparents, and thus there may have been only weak selection against specific or nonspecific accumulation of these elements. We also found more QTL than expected for nonessential analogs (1.99×, binomial test *P *=* *0.002). The nonessential analog Sr was phenotypically correlated with its chemical analog Ca at every garden, and they shared colocalized QTL at the two large clusters on Chr02N (at the top) and Chr07K in our cross. Strong correlations between Sr and Ca have been reported in other species (Broadley and White, 2012; Shakoor *et al.* 2016). The colocalization of QTL of Sr with other elements also likely reflects its nonessential nature, in that it is seldom the target of uptake by plants, and instead only accumulates via nonion-specific mechanisms.

We found multiple candidate genes within our QTL regions which may affect element concentrations. These candidate genes provide targets for future fine-mapping research in switchgrass. Among these, we found a homolog of *HKT1*, *Pavir.7kg416470*, in the QTL on Chr07K. This candidate gene was in the QTL interval for the six elements, Al, Ca, Fe, Mn, Sr, and Zn, but not in either of the two Na accumulation QTL intervals. *HKT1*, which encodes Na transporter, was responsible for the variation in Na accumulation in *A. thaliana* (Rus *et al.* 2006; Baxter *et al.* 2010), rice (Ren *et al.* 2005; Kobayashi *et al.* 2017), wheat (Munns *et al.* 2012), and maize (Zhang *et al.* 2018). However, Na accumulation in these studies were assayed in plant leaves, while Na accumulation in our study was assayed from whole tillers, which included both leaves and shoots. It seems likely that different tissues could accumulate elements at different levels, but our data represents a composite picture of several tissues. In addition, soil Na was not particularly variable in our gardens (*i.e.*, 11, 12, and 10 ppm for TX, MO, and MI, respectively), and some of these elements do compete with Na uptake from soil (Mass *et al.* 1972; Cramer *et al.* 1989; Tuna *et al.* 2007). It is also possible that the lack of variability of soil Na relative to these other elements masked a QTL effect for Na but allowed detection of this QTL for other elements.

Overall, our results suggest that ionomic variation, and ionomic variation across environments, are common in switchgrass. This variation, controlled by a combination of genes and the environment, offers critical material for adaptation of switchgrass metabolism and development across different environments. The identification of loci that affect nutrient concentration in these environments will facilitate the development of switchgrass varieties with high nutrient-use efficiency for sustainable biofuel production. When combined with harvested biomass, plant elemental concentrations can be linked to nutrient removal from the soil and impact biofuel conversion efficiency and future soil fertility.
